# A Comparative, Individual Values-Based Scoring Approach to the Secure Flourish Index Among Clinical Health Professions Students

**DOI:** 10.1007/s40670-024-02182-x

**Published:** 2024-11-07

**Authors:** Stephanie Neary, Benjamin Doolittle, Martina Mueller, Michelle Nichols

**Affiliations:** 1 Yale University, PO Box 208004, New Haven, CT 06520-8004 USA; 2 College of Nursing, Medical University of South Carolina, Charleston, SC USA; 3 Department of Public Health Sciences, Medical University of South Carolina, Charleston, SC USA

**Keywords:** Flourishing, Student well-being, Thriving, Medical education, Secure Flourish Index

## Abstract

**Purpose:**

The purpose of this study was to investigate flourishing among medical (MD), physician assistant (PA), and nurse practitioner (NP) students, using the novel Secure Flourish Index (SFI).

**Method:**

MD, PA, and NP students from two institutions completed the traditional SFI (tSFI), then applied a percentage weight to each of the six domains (maximum total 100%) based on perceived relative importance to their overall flourishing, creating a novel self-weighted SFI score (swSFI). The Bland–Altman (BA) plot was used to assess the magnitude of agreement between scores.

**Results:**

The BA plot (n = 281) revealed a mean bias of .07(95% CI -.50,.63). Eighteen participants (6.4%) fell outside of the calculated BA limits of agreement [-9.31 [95% CI - 10.27,-8.45] and 9.45 [95% CI 8.49,10.41]]. Linear regression revealed the mean BA score is predictive of the mean difference between scores [R^2^ = 0.07, F(1,280) = 21.1, p < .001] indicating bias in agreement between the scoring systems as mean flourishing score changes.

**Conclusion:**

Accounting for individual values is important when measuring student flourishing but is missing from current operant definitions. The overall mean difference (bias) in tSFI and swSFI scores is minimal (.07, possible range 0–120). However, the bias becomes larger as individual mean flourishing scores move towards both the high and low ends of the flourishing spectrum. This indicates that the influence of weighting flourishing domains is larger for individuals with high or low flourishing than those with moderate flourishing.

**Supplementary Information:**

The online version contains supplementary material available at 10.1007/s40670-024-02182-x.

Decreased mental health is a leading cause of attrition among students training in health professions [1, 2]. These students often experience mental illness, burnout, and suicidal ideation at higher rates than the general population [3–6]. Historical approaches to resolving the mental health crisis facing medical (MD), physician assistant (PA), and nurse practitioner (NP) students have been primarily deficits-based, focused on mitigating burnout rather than on the promotion of flourishing [7, 8]. While important, waiting until students are suffering from burnout before intervening fails to capture foundational skills needed to flourish and lead a fulfilling, purpose-driven life [9].

Flourishing can be difficult to define as the concept is inherently interdisciplinary, incorporating aspects of psychology, sociology, and theology [10]. Flourishing is a transient life course, not a fixed state of being, and can be highly influenced by individual, interpersonal, and organizational factors [11]. Conceptually, it has roots in Aristotle’s description of eudaimonia, the idea that one’s life should have elements of both virtue and pleasure [12]. Living in accordance with individual meaning and purpose is a core domain of human flourishing. However, an individual’s perception of flourishing is shaped by both individual and environmental factors [10].

Multiple intrinsic characteristics influence personal definitions of flourishing including age, gender, and mental health status as well as general positive or negative affect [13–15]. However, it has been found that the intersection of mental illness and flourishing does not follow a uniform trajectory across the lifespan, and little is known how intensive medical and nursing training programs further influence these patterns [16, 17]. Younger adults tend to find more meaning in personal growth, which has also been observed in medical students who often knowingly defer short-term gratification for long-term academic and professional success [11]. Positive affect is related to increased flourishing among medical students and has been found to have greater influence over both satisfaction and individual meaning than negative affect [14, 18, 19].

The structure of medical education systems can either help or hinder student flourishing. Integrated wellness efforts that include faculty participation have been shown to increase student flourishing while required participation in low-value obligations and a lack of recovery time between high commitment activities reduce student flourishing [11]. For example, incorporating faculty mentorship into the curriculum is higher yield than mandatory webinars students must complete on their own time [11]. Despite known preference of integrated wellness efforts, there remains limited consensus on how to best measure flourishing among healthcare students.

Vanderweele et al. developed a concept of human flourishing that acknowledges contributions of positive psychology, virtue ethics, and overall well-being [10]. His domains, which can be measured using the Secure Flourish Index (SFI), include Happiness and Life Satisfaction, Mental and Physical Health, Meaning and Purpose, Character and Virtue, Close Social Relationships, and Financial and Material Stability [10]. However, this definition assumes that each domain is valued equally among all individuals. There is a clear need to take an individualized, strengths-based approach to addressing student need. Further exploration among students is needed to better understand how individual values, demographics, and life experiences affect flourishing [13]. At the organizational level, understanding these factors has the potential to aid in the development of more effective and personalized interventions, which could positively impact student attrition and burnout rates. The purpose of this study was to determine the level of agreement between flourishing scores when evaluated using the Secure Flourish Index traditional scoring approach versus a novel, individual values-based weighted scoring approach among MD, PA, and NP students.

## Method

### Setting, Sample Population, and Recruitment

Students enrolled in MD, PA, and NP programs from two US academic medical centers were recruited to participate in this cross-sectional study. One institution is private and located in the Northeast and the other is public and in the South. Both institutions have traditional campus-based MD programs and a combination of online or hybrid and/or campus-based PA and NP programs. MD-PhD and PA-MPH students were included. Additionally, while nursing students pursuing either a masters (Master of Science in Nursing) or doctoral degree (Doctor of Nursing Practice) were included, PhD in Nursing Science students at both institutions were excluded as their programs are not clinical in nature. Similarly, students in other clinical degree-seeking programs were excluded (e.g., dental, pharmacy, midwifery) given the narrower scope of patient care. Students across all years in the identified programs were included. A target response of 318 was calculated using a 95% confidence interval with a 5% margin of error based on a population size of 1820 [20]. Survey recruitment occurred between August 6 and October 9, 2023, through a combination of emails, flyers, and live in-person and online sessions.

### Survey Design and Instrument Scoring

Vanderweele’s Secure Flourish Index (SFI) has operationalized his concept of human flourishing, offering a method to begin to quantify this complex concept [10]. The traditional SFI scoring approach provides zero to ten points for each of 12 questions, divided into six domains of two questions each: Happiness and Life Satisfaction, Mental and Physical Health, Meaning and Purpose, Character and Virtue, Close Social Relationships, Financial and Material Stability. This produces an overall flourishing score with a possible range of 0–120 and six domain-specific flourishing scores with possible score ranges of 0–20 each. While providing a strong foundation for the assessment of flourishing, individuals may value the relative importance of each of these six domains differently, which is not accounted for in the current scoring approach that equally weights the relative value of each domain [13, 21].

For example, two individuals may both score a question on being content with their current social relationships a 7/10. However, one individual may not believe that social relationships strongly influence their ability to flourish while the other may believe that social relationships are foundational in flourishing. With the traditional scoring method, both individuals receive a score of 7/10, but this does not reflect the individual, perceived importance of social relationships to their ability to flourish. This study proposes an alternative scoring method where the participant provides relative domain weights totaling 100% across the six domains to create a new, self-weighted SFI score taking into account the individuals’ perceived importance of each domain (Table 1).

**Table 1:** Example of Self-Weighted Secure Flourish Index (swSFI) scoring as compared to traditional scoring (tSFI) with conversion calculation

Domain	Question 1 score	Question 2 score	Domain average score	Assigned weight %	swSFI domain score^a^	tSFI domain score^b^
Happiness and Life Satisfaction	8	7	7.5	10	.75	1.25
Mental and Physical Health	7	7	7	10	.70	1.17
Meaning and Purpose	6	10	8	45	3.60	1.33
Character and Virtue	10	10	10	15	1.50	1.67
Close Social Relationships	6	5	5.5	5	.28	.92
Financial and Material Stability	9	9	9	15	1.35	1.50
*Total score out of 10*					8.18	7.84
*Conversion*					**8.18/10 = *x*/120**	**7.84/10 = *x*/120**
*Total score out of 120 possible points*					**98.2**	**94.1**

^a^Self-weighted domain sum score is calculated by multiplying the average domain score by the weighted percentage value assigned by the participant

^b^Traditional domain weight sum score is calculated by multiplying the average domain score by 16.667% (calculated by dividing 100% evenly across the six domains)

Survey data were collected and managed using REDCap electronic data capture tools hosted at the Medical University of South Carolina [22]. Students who agreed to the statement of research were asked to complete a series of validated instruments and demographic questions. Participants completed the SFI and then were asked to apply a relative percentage weight (0–100%) to each of the established six flourishing domains based on their perceived relative importance to their individual flourishing [10]. Domain weights (percentages) were then applied to SFI responses to produce a novel, self-weighted SFI score. The established scoring approach will be referred to as the traditional approach (tSFI) and the novel approach will be referred to as the self-weighted approach (swSFI). An example of the comparative scoring approaches, where the participant assigns different weights for each domain, is outlined in Table 1.

Using this example, the self-weighted SFI scoring approach results in a flourishing score that is four points higher than the traditional scoring approach. It also reveals that this individual placed a much higher relative value on Meaning and Purpose (45.0%) than on Close Social Relationships (5.0%).

### Missing Data

Cases missing more than one tSFI response(s) were filtered out of the data set to avoid making multiple assumptions regarding the missing values. For cases missing an entry for one question in the tSFI, the score was imputed by duplicating the response for the second question in the domain for the missing score. For example, if question one was missing a response, the score from question two (also within the domain of Happiness and Life Satisfaction) was used as the score for the missing question one data. Following imputation, participant data was included only if the participant also completed the self-weighed SFI sections completely and correctly (swSFI total must equate to 100%), as complete responses were needed to accurately calculate scores (Table 1). Additional cases with missing data for demographic questions were not included in the respective analysis involving these questions; however, they were included in analyses of other questions and instruments that had complete data. The complete survey can be found in Appendix 1. 

### Statistical Analysis

Descriptive statistics were used to summarize demographic characteristics, including means, standard deviations, medians, and interquartile ranges when applicable. The Bland–Altman (BA) plot was used to determine the agreement between the tSFI and the swSFI scoring approaches [23]. Bland–Altman plot graphs the difference (bias) between the methods (traditional SFI score − self-weighted SFI score) against the mean flourishing score (traditional SFI score + self-weighted SFI score)/2 [23]. Acceptable limits of agreement (LoA; precision) were not set a priori given the exploratory nature of the project and post hoc judgement was used to determine sufficiency of the LoA with 95% confidence limits (mean difference ± 1.96). The assumption of normality was met for each the tSFI, swSFI, and calculated differences in scores through visualization of approximate normal distribution using histograms. The Preiss-Fisher [24] procedure was conducted with 100 random pairings of the tSFI and swSFI scores to verify the data range was sufficiently wide to conduct the BA test, supporting that the results are unique to the pairing of the original data set and BA plots are appropriate for analyses. Data sets with small ranges may produce practically acceptable precision results secondary to any pairing of the data points, removing the value of the BA test. Surveys were only obtained once from each participant; therefore, analysis of repeatability is not applicable. A scatterplot was used to plot the mean differences (bias) on the vertical axis against the mean of the flourishing scores on the horizontal axis. Linear regression was carried out to identify trends across measurement sizes, addressing whether the agreement between the two scoring approaches varies when mean flourishing scores are low versus high [25]. The analysis was repeated using the natural log of the scores to further confirm the results.

The cases lying outside of the LoA were filtered from the data set and descriptive statistics were obtained for the outliers (*n* = 18), with the adjusted primary data set without the outliers (new *n* = 263), to investigate whether participants in these groups differed in terms of demographic characteristics. Means were compared using the Kruskal–Wallis test due to the unequal and small sample sizes. Data were analyzed using Statistical Package Social Sciences (SPSS) version 28 (IBM; Armonk, NY) and Microsoft Excel Version 16.48; significance was set at alpha of 0.05.

The study was approved by the Institutional Review Boards at the Medical University of South Carolina (protocol #00129125) and Yale University (protocol #2000035757).

## Results

Of the estimated 1820 eligible students, 390 (21.4%) students began the survey. A total of 281 (281/1820; 15.4%) students completed the tSFI and the swSFI questions with a total weight across the six domains of 100% and were included in the analysis. A total of 45.2% were PA students (*n* = 127), 33.8% of participants were MD students (*n* = 95), and 21.0% were NP students (*n* = 59). Students spanned from recent matriculation (< 3 months) through year four, the average age of participants was 28.7 years (SD 6.6), and 75.1% (211/275) identified as female (Table 2).

**Table 2:** Medical, physician assistant, and nurse practitioner student demographic and descriptive data (*N* = 281)

	*n*	*M (SD); median (Q1:Q3)*
Age, years	269	28.7 (6.5); 26.0 (24.0:32.0)
Number of legal dependents	264	0.5 (1.0); 0.0 (0.0:0.0)
	***n***	**(%)**
Profession (*n* = 281)		
*Medical Doctor (MD)*	95	33.8
*Nurse Practitioner (NP, DNP)*	59	21.0
*Physician Assistant/Associate (PA)*	127	45.2
Gender Identity (*n* = 275)		
*Man*	61	22.2
*Woman*	211	75.1
*Other/Prefer not to disclose*	2	1.1
Race (*n* = 281)^a^		
*White*	197	70.1
*Black or African American*	17	6.0
*American Indian or Alaskan Native*	5	1.8
*Asian*	49	17.4
*Native Hawaiian or Pacific Islander*	0	0.0
*Prefer not to answer*	17	6.0
Hispanic, Latinx, or Spanish (*n* = 274)		
*Yes*	16	5.8
Year in training (*n* = 275)		
< *3 months*	59	21.5
*3 months–1 year*	67	24.4
*Year 2*	91	33.1
*Year 3*	32	11.6
*Year 4*	26	9.5
Relationship status (*n* = 275)		
*Single*	172	62.5
*Married/Domestic Partner/Civil Union*	96	34.9
*Separated*	2	0.7
*Divorced*	4	1.5
*Widowed*	1	0.4
Parental Undergraduate Degree Status (*n* = 274)		
*Yes, both parents*	158	57.7
*Yes, one parent*	52	19.0
*No, neither parent*	64	23.4

^a^Asked as a “select all that apply” therefore total percentage equates to more than 100% as some participants selected more than one category

### Flourishing Scores

The mean tSFI score was 84.1 (SD 15.0) and the mean swSFI score was 84.0 (SD 16.3). The domains of Meaning and Purpose and Character and Virtue had the highest mean scores with 15.3 (SD 3.5) and 15.2 (SD 2.7) respectively on the tSFI, but participants weighted Character and Virtue as the domain with the lowest relative contribution to their perceived ability to flourish (13.2% (SD 6.5)). The domain of Financial and Material Security had the lowest mean score of 12.7 (SD 5.4) and the second lowest relative contribution to perceived flourishing (14.7% (SD 8.6); Table 3).

**Table 3:**
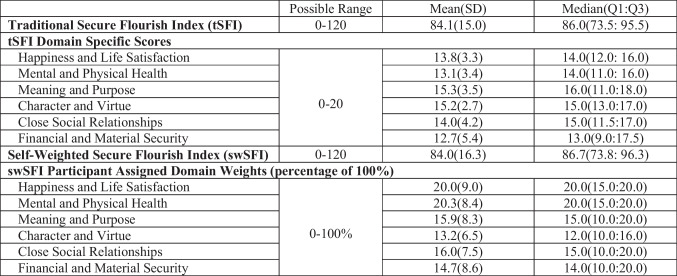
Mean flourishing scores using the traditional Secure Flourish Index and the novel Self-Weighted Secure Flourish Index approaches (*N* = 281)

### Bland–Altman Plot

**Fig. 1:**
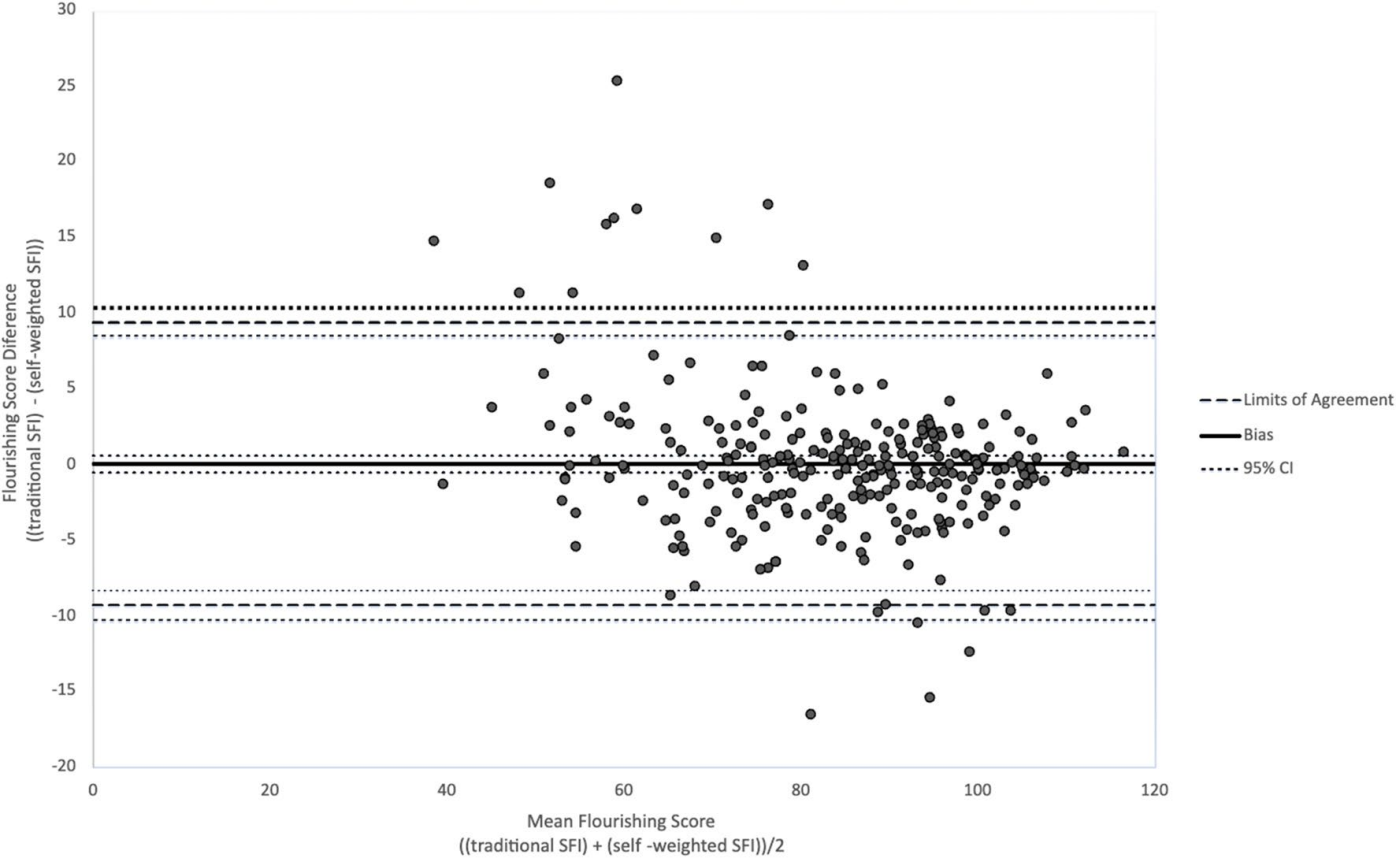
Bland–Altman plot of flourishing score difference and mean flourishing score

The bias, calculated from the estimated mean differences, was 0.07 with a 95% confidence interval of − 0.50 to 0.63. This is interpreted as showing that on average, swSFI scores were 0.07 points higher than tSFI scores for a given participant. The limits of agreement (BA LoA) were − 9.31 [95% CI − 10.27, − 8.45] and 9.45 [95% CI 8.49, 10.41], as shown in Fig. 1. The Preiss-Fisher procedure showed the original *R*^2^ (0.96) falls outside of the mean ± 2 standard deviations of all 100 mismatched pairings of *R*^2^ (0.01). This indicates there was a sufficiently wide measurement range as less than 5% of all random pairing combinations met the acceptable limit for precision, ruling out that precision could be met secondary only to chance. The data structure was considered when deriving the 95% confidence intervals of the BA LoA.

The BA plot (Fig. 1) shows a variation in agreement as the measurement varies (more positive difference with lower mean score and a more negative difference with higher mean scores), the mean difference was regressed on the mean of the two scores as recommended by Bland and Altman [25]. This revealed that the mean BA score is predictive of the mean difference between the scores [*R*^*2*^ = 0.07, *F*(1, 280) = 21.1, *p* < 0.001] and the null hypothesis that there is no difference (bias) in the level of agreement between the two scoring systems was rejected. On average, the swSFI reported a score 0.08 points higher for each point increase in the tSFI score [*predicted mean difference* = *6.9 − 0.8 (mean flourishing score)*]. To further verify the findings of disagreement, regression was repeated with the un-imputed data (the five cases with a missing tSFI question were excluded) and results were similar. Additionally, regression using the natural log of the mean difference and mean flourishing scores to reduce the potential effect of variance on the outcome provided similar results (*p* < 0.001).

### Outliers

Eighteen of 281 cases (6.4%) fell outside of the LoA. Typical LoA support agreement when < 5.0% of cases are outliers [26]. When comparing mean tSFI scores for cases within and outside the LoA, outliers have lower means compared to cases within the LoA: mean tSFI was 75.5 (SD 15.1) vs 84.6 (SD 14.9) (*p* = 0.02) and mean swSFI score was 70.7 (SD 26.5) vs 84.9 (SD 15.0) (*p* = 0.04). For tSFI, the largest difference in individual flourishing domain means was in the category of Close Social Relationships when comparing those within (14.2 (SD 4.1)) and outside (11.1 (SD 5.7)) of the LoA (*p* = 0.03). When comparing domain weights on the swSFI, the LoA outlier mean Character and Virtue domain weight was 6.2% (SD 4.8) compared to a mean of 13.6% (SD 6.3) for those within the LoA (*p* ≤ 0.001). Students in the LoA outliers group were an average of 3.6 years older (32.0 (SD 7.6)) than those in the within the LoA (28.4 (SD 6.4)) (p = 0.04; Table 4). Additionally, 61.1% of students in the LoA outliers group were PA students, as compared to 44.1% of those within the LoA.

**Table 4:**
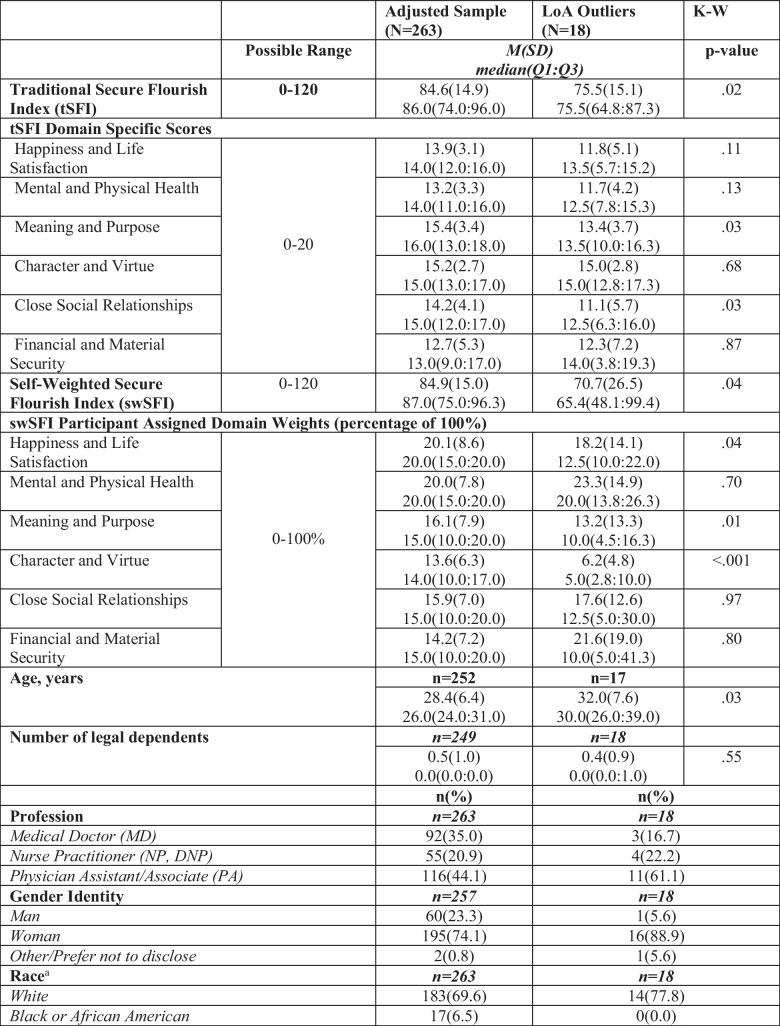

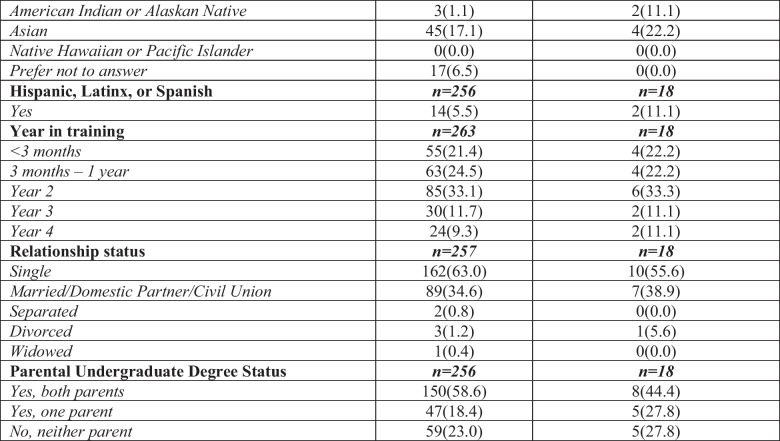
Comparison of mean tSFI and swSFI scores between participants within the limits of agreement and outliers (*N* = 281)

^a^Participants asked to “select all that apply” so percentage equates to more than 100% as some participants selected more than one race

## Discussion

Pursuing a strengths-based, flourishing-centric approach to the mental health crisis facing healthcare providers fosters personal development alongside clinical acumen. Promoting a learning environment that invests in personal formation, not only clinical knowledge, may encourage students towards flourishing. Medical and nursing education has focused on competency as the primary measure of success [27–29]; by meeting the minimum threshold score, students are advanced through training without regard to their overall well-being. Students are aware of this vertical hierarchy of clinical disciplines and admit to trading personal wellness for academic success through training [11]. However, success as a provider depends as much on trust and relationship building as it does academics, but these skills are much more difficult to evaluate. Incorporating individual values into the measurement of flourishing through the swSFI introduces a framework for identifying potential non-academic drivers and barriers to success as a provider. To our knowledge, this is the first study to incorporate individual value-based weights to the SFI.

The swSFI approach presented here is not intended to replace the tSFI as a negligible score difference resulted for most students. However, for those with larger mean differences, the swSFI may more accurately reflect their overall flourishing; and the regression model shows this is more likely to occur for those on the very high or very low end of the flourishing spectrum. Additionally, there are currently no established cutoffs in the tSFI domains to determine human flourishing. However, a mean score above 7 (0–10) in the Happiness and Life Satisfaction domain has been used in prior studies to define human flourishing as it correlates with the multi-item, widely used Satisfaction with Life Scale [30–32]. These findings suggest the establishment of such a cutoff score value may not be equivalently representative of individual flourishing for all students. Gaining insight of what aspects of flourishing students most value, including how cultural values and practices may influence perceptions of flourishing, may provide an avenue for a more individualized approach to wellness efforts.

### Expanding Support

Asking participants to weight the different domains of flourishing may also provide insight into a more personalized approach to support for students. Understanding what individual students value challenges faculty leaders to expand our definition of support beyond academics and seek ways to foster individual development. Rather than relying on point-in-time wellness initiatives such as yoga classes or meditation breaks, the swSFI shows that wellness activities may have a relative impact among students. Professional development workshops may have a larger impact on students who place a higher value on the domain of Character and Virtue while expanded access to exercise facilities and telehealth options may have a larger impact on those who place higher value on Mental and Physical Health. Similarly, while not statistically significant, it is clinically relevant that students outside the BA LoA perceived the relative importance of Financial and Material Stability in flourishing to be much higher than those within the LoA (21.6% vs 14.2%); further research is needed to determine how a student’s current financial situation impacts their perception of this domain and overall flourishing.

### Strengths and Limitations

Strengths of this study include the use of a widely validated instrument and the inclusion of students from multiple degree programs at two separate institutions. Weighing each domain of the tSFI gives a novel insight into individual values among trainees and broadens the understanding of human flourishing. Additionally, the reliability of the precision of the BA test was checked using the Preiss-Fisher procedure; the BA results were confirmed using the natural log transformation of the data.

This study has multiple limitations, including a sample size below the calculated target. This was a result of the reduced ability to recruit medical students from one institution as it was going through Liaison Committee on Medical Education accreditation and did not approve the survey for distribution via email so as not to interfere with accreditation-related surveys. NP students from one institution were approved to participate, but approved recruitment methods only included flyers at a distant campus for primarily off-campus students, which resulted in no participation. Doctor of Osteopathic Medicine students were not included in this study as neither institution currently offers this degree program. However, these individuals are pursuing a degree of similar caliber, length, and scope of practice and including this population in future studies could provide valuable insight. Additionally, individuals who have higher social needs may have had increased incentive to participate as participants were provided a five-dollar gift card. Conversely, the discussion of sensitive and personal topics such as finances and mental health may have dissuaded participation from students facing these challenges. Finally, as participants created their own unique identifier, participants could have completed the survey more than one time and agreement would be affected by the natural variation inherent to the rater.

### Future Directions

These findings can be used to establish a priori limits of agreement for future studies in expanded populations as a first step towards validating the swSFI as a measure of flourishing. Based on the differences in agreement found in this population, we believe it is reasonable to establish clinically acceptable LoA of ± 6 for future studies. This is determined by allowing for an average of a 5% (1 point) difference in flourishing score across each domain, which we believe to be clinically relevant. Further research is needed to explore the role of demographics and the social determinants of health in the weighting of flourishing domains and how these factors may be related to differences between tSFI and swSFI scores to better interpret a mean difference in flourishing scores that is clinically relevant. Additionally, clinical relevance may differ based on overall flourishing score, as shown in this study. The differences in multiple flourishing domain average scores for those outside the LoA show the importance of exploring and accounting for how individual values influence scoring outcomes in future efforts to operationalize flourishing. Additionally, while not statistically significant, it is clinically relevant that students in the LoA outliers group place more value on Financial and Material Stability highlighting a possible intersection of social needs and flourishing.

## Conclusion

Accounting for individual values is an important piece of measuring student flourishing that is missing from current operant definitions. While the mean difference in tSFI and swSFI scores is minimal (0.07), individuals who fall on either the high or low end of the flourishing spectrum experience more bias between the scoring approaches than those who have more medially aligned scores. Self-weighting the domains may produce a higher overall flourishing score than the tSFI for individuals with higher mean flourishing and a lower overall flourishing score for individuals with lower flourishing. This shows that the current scoring approach may be overestimating flourishing for individuals who have low flourishing and underestimating flourishing for individuals with high flourishing. Further exploration of how student demographics, life experiences, and values influence perceived relative weights of flourishing domains is needed to promote learning environments that foster individual flourishing.

## Supplementary Information

Below is the link to the electronic supplementary material.

Supplementary file1 (PDF 968 KB)
